# Supplementary material for: Analyzing green and sustainable land use in China’s coal cities: Insights from industrial transformation
Source: PLoS One. 2024 Apr 24;19(4):e0301836. doi: 10.1371/journal.pone.0301836 (PMC11042707; doi:10.1371/journal.pone.0301836)
Supplement: Attachment — Submitted filename: Response to Reviewers#2.docx [file pone.0301836.s001.docx]

**Response Letter**

Dear Editors and reviewers :

Thank you for your letter and comments concerning our manuscript entitled

We sincerely thank the editor and all reviewers for their valuable feed-back that we have used to improve the quality of our manuscript. The reviewer comments are laid out below in italicized font and specific concerns have been numbered. Our response is given in normal font and changes/additions to the manuscript are given in the red text.

*Reviewer #2 1- Please read and check the paper thoroughly as there are sentences which has been found to be redundant (line 47).*

Author’s reply

We sincerely thank the reviewer for careful reading. As suggested by the reviewer, we have rechecked the redundant sentences and rewritten the section (line 41-42).

*Reviewer #2 2-To re-write the sentence from line 65-69 as they are rather confusing.*

Author’s reply

We feel sorry for our carelessness. In our resubmitted manuscript, We have re-written this part according to the Reviewer's suggestion (line 71-77). Thanks for your correction.

*Reviewer #2 3- Figure 1 (line 217) should be placed under the method section as it prematurely depicts the method used (the model and the applied regression test).*

Author’s reply

We think this is an excellent suggestion. We have adjusted the position of Figure 1, the new position is after the research method Tobit regression section in the revised version (line 335-336).

*Reviewer #2 4-Suggestion to move the study limitations (line 559) to the early part of the paper and not under the discussion section.*

Author’s reply

We think this is an excellent suggestion, we appreciate the constructive feedback. The revisions aim to enhance the completeness of our analysis, the research limitations are briefly presented in the INTRODUCTION section of the research and expanded again in the DISCUSSION, in order to point out the research limitations and potential avenues for future research in detail.

Thanks again to the reviewers and editors for their valuable comments and suggestions on this article, which further improves the quality of the paper! We have learned from your valuable comments to improve the level of article writing experience and the spirit of excellence. If there is anything wrong with this article, please inform us, we will continue to improve and modify, thank you very much!
