# Supplementary material for: Analyzing green and sustainable land use in China’s coal cities: Insights from industrial transformation
Source: PLoS One. 2024 Apr 24;19(4):e0301836. doi: 10.1371/journal.pone.0301836 (PMC11042707; doi:10.1371/journal.pone.0301836)
Supplement: Attachment — Submitted filename: Response to Reviewers#1.docx [file pone.0301836.s002.docx]

**Response to Reviewer #1**

Dear Reviewer #1 :

We would like to thank you for your professional review work, constructive comments, and valuable suggestions on our manuscript.

We wish you all the best in your work!
